# Advances in the etiology and neuroimaging of children with attention deficit hyperactivity disorder

**DOI:** 10.3389/fped.2024.1400468

**Published:** 2024-06-10

**Authors:** Fang Shen, Hui Zhou

**Affiliations:** Department of Pediatrics, West China Second University Hospital, Sichuan University, Key Laboratory of Birth Defects and Related Diseases of Women and Children of Ministry of Education, Chengdu, China

**Keywords:** attention deficit hyperactivity disorder, genetics, environmental risk factors, epigenetics, neuroimaging features, further research directions

## Abstract

Attention deficit hyperactivity disorder (ADHD) is the most common neurodevelopmental disorder in children, characterized by age-inappropriate inattention, hyperactivity, and impulsivity, which can cause extensive damage to children's academic, occupational, and social skills. This review will present current advancements in the field of attention deficit hyperactivity disorder, including genetics, environmental factors, epigenetics, and neuroimaging features. Simultaneously, we will discuss the highlights of promising directions for further study.

## 1 Introduction

Attention deficit hyperactivity disorder (ADHD) is a prevalent neurodevelopmental disorder, characterized by extensive hyperactive, impulsive and/or inattentive behaviors that impair daily functioning. Notably, it is estimated the global prevalence of ADHD as 7.2% (1, 2). And in China specifically, ADHD affected around 6.4% of children (3). Long-lasting clinical symptoms are present in between 50% and 60% of ADHD patients, frequently coexisting with additional disorders, namely anxiety, oppositional defiant disorder, and tic disorders. These conditions can increase the risk of suicide and delinquency and have a major negative influence on families and society (4, 5). The complicated etiology of ADHD is frequently attributed to a confluence of hereditary and environmental variables (6, 7). Research has demonstrated that structural abnormalities in the brain, such as decreased brain volume and cortical surface area in ADHD, can cause disturbances in brain function, which can result in executive dysfunction and a variety of clinical symptoms, including impulsivity, hyperactivity, and inattention (7, 8).

## 2 The etiology and pathogenesis of ADHD

Although the exact cause of ADHD remains unidentified, most researchers contend that it is a result of a combination of hereditary and environmental factors. Besides, ADHD has a comparatively variable clinical presentation, by thoroughly examining its various etiology and pathogenesis, new treatment approaches can be developed and researched more easily.

### 2.1 Genetic factors

The development of ADHD is largely influenced by genetic factors, and investigations involving twins and family lines have demonstrated a distinct familial clustering in the disorder's development. Additionally, research conducted by Uchida et al. (9) found that children whose parents had ADHD were more likely than their non-ADHD parents to experience ADHD and other cognitive and psychiatric disorders. Faraone et al. demonstrated that the heritability of ADHD in twins is approximately 74% (10). Through a genome-wide association study (GWAS) of ADHD, Demontis et al. (11) presented a significant research paper in 2019 which achieved substantial progress in identifying genetic risk factors and refining the genetic architecture of the disease. The research revealed 12 independent genome-wide significant loci associated with ADHD by combining a genome-wide association meta-analysis of ADHD risk genes with data from the Danish Integrative Psychiatric Research (iPSYCH) and 11 ADHD cohorts from various nations that were gathered by the Psychiatric Genomics Consortium (PGC), totaling 20,183 ADHD patients and 35,191 controls.

Primarily, the identified risk genes are clustered in the cerebral cortex and are associated with early brain development, principally involving several brain-specific neuronal subtypes and midbrain dopaminergic neurons. The risk gene loci are linked to neurodevelopment, neurotransmitter transmission, and regulation of gene expression. The aforementioned study provides compelling evidence that ADHD is a polygenic disorder, with several risk genes working together to determine the development of the condition and varying degrees of illness risk associated with each gene. In 2023, Demontis et al. identified 27 risk loci, comprising both the common loci from the 2019 analysis and recently found rarer genetic loci. Simultaneously, after examining the expression of ADHD risk genes in different tissues and cell types, the researchers discovered that many of these genes were enriched in excitatory and inhibitory brain neuronal cell types. Additionally, it was demonstrated that genes expressed in dopaminergic mesencephalic neurons and genes related to ADHD were significantly correlated (*P* = 0.005) based on the results of single-cell RNA sequencing data and cell type-specific analysis (12).

Furthermore, the ongoing advancement of research on disease risk gene loci (13) will gradually lead to the genetic elucidation of the effects of risk genes on the development of particular neuronal subtypes and brain network connections, as well as the further clarification of the connection between attention-deficit, hyperactivity, and impulsive behaviors in children with ADHD and abnormalities in brain development. Based on prior research, an estimated 300 candidate genes have been linked to the onset of ADHD. These genes are primarily found in midbrain interneurons. We will then describe in detail three common interneurons including dopaminergic neurons, noradrenergic neurons, and 5-hydroxytryptamine (5-HT) neurons.

#### 2.1.1 Association of dopaminergic genes, dopamine neurotransmission pathway and ADHD symptoms

##### 2.1.1.1 Dopamine receptor genes

Dopamine receptor D4 (DRD4) is the most studied candidate gene associated with increased risk of ADHD, DRD4 receptor regulates dopamine signaling in the CNS and plays important roles in attention, reward, and motivation. Chang et al. (14) found that children with the DRD4 GG genotype were more likely to experience ADHD than children with the DRD4 GA/AA. Notably, variable Number Tandem Repeats (VNTR) is a repeated section of the DNA sequence that is the main target of variations in the DRD4 gene. The 7 repeat (7R) and the 4 repeat (4R) are two of the most prevalent variation types. Research has identified a correlation between the 7R variation and a higher likelihood of developing ADHD. According to some research, those who have the DRD4 7R variation may be more prone to ADHD in specific groups. However, the results are inconsistent, as the association's intensity and direction changed based on populations and research. Thus, the DRD4 gene's connection to ADHD remains debatable, and other genetic and environmental variables may influence how the gene functions. The relationship between DRD5 gene variations and ADHD has not been researched adequately. Despite this, Tong et al. (15) have suggested that some variations in the DRD5 gene may be associated with an increased chance of developing ADHD. Whereas other research has suggested that the DRD5 gene and ADHD do not appear to be correlated. Therefore, to validate the DRD5 gene's involvement in the pathogenesis of ADHD, additional research is required.

##### 2.1.1.2 Dopamine transporter genes

An increased risk of ADHD has also been linked to variations in dopamine transporter genes, such as those in the dopamine transporter 1 (DAT1, also known as *SLC6A3*) gene. One of the most extensively researched potential genes in the pathophysiology of childhood ADHD is DAT1 (16). Research has indicated that the 10R of the 40 bp VNTR in the 3′ untranslated region (3′UTR) of DAT1 is strongly correlated with clinical symptoms in ADHD children, particularly those with attention deficits and that DAT1 haplotypes comprising the 10R of the 40 bp VNTR of the 3′UTR/the 6R of the 30 bp VNTR of the intron 8 are strongly correlated with cognitive impairments in ADHD. The 10R/10R genotype of DAT1 VNTR is related to ADHD in Korean children (17). Study in Jordan children showed that the 10R allele of *DAT1* was associated with ADHD in the children (18). On the other hand, conflicting results were reported in the studies in the Omani children, Han Chinese children, Iranian population and Turkish population which showed no association significant between VNTR polymorphism with ADHD (19–22). Meanwhile, it has been reported that there is no association between VNTR polymorphism of *DAT1* genes and ADHD among Indonesian children based on a case-control study (23). In 2020, Kuc et al. also found that the *SLC6A3* gene polymorphism was not associated with the presence of ADHD (24).

#### 2.1.2 Genes associated with the 5-hydroxytryptaminergic system

The frontal orbital-striatal pathway is regulated by 5-hydroxytryptamine (5-HT), and 5-hydroxytryptamine receptors (5-HTR) are mainly located in the brain's prefrontal cortex, amygdala, and hippocampus (25). Deviations from normalcy in these brain regions have a substantial impact on children with ADHD's hyperactivity, impulsivity and attention span. The presence of two crucial 5-HT receptors, specifically 5-hydroxytryptamine 1A and 2A receptors, have been identified as closely related to the behaviors associated with ADHD (26). Additionally, the 5-hydroxytryptamine transporter (SERT), 5-HTR 1B and 5-HTR 2A genes are involved in the pathophysiology of ADHD (27). The 5-hydroxytryptamine transporter gene (SLC6A4/5-HTT) encodes the 5-hydroxytryptamine transporter (SERT) and regulates the effectiveness of 5-HT. This gene promoter region (5-HTTLPR) is associated with ADHD pathogenesis as the long allele (L) and the short allele (S). Children with ADHD exhibit more evident behavioral issues and hyperactivity when they have the “S” allele and the “S/S” genotype, and a greater degree of executive function impairment when they have the “L” allele (28).

#### 2.1.3 Genes connected to the noradrenergic system

The norepinephrine transporter (NET, SLC6A2) was discovered to reuptake sympathetically released norepinephrine (NE) into the presynaptic membrane via active transport. Since norepinephrine transporter antagonists can affect the effectiveness of pharmacological treatment in children with ADHD, the norepinephrine transporter gene has emerged as the most extensively researched noradrenergic system gene. According to Shang et al. (29), children with ADHD can have their intrinsic brain activity, attention, and visual memory regulated by the norepinephrine transporter gene. Shirama et al. (30) concluded that NE modulation of serum concentrations and gene interactions alter alertness in ADHD patients, leading to an increased likelihood of dangerous behaviors in ADHD patients. The findings of Hawi et al. (31) revealed a high correlation between the development of ADHD and SLC6A2. Wang et al. (32) found that the α-2A adrenergic receptor (ADRA2A) gene is also a candidate gene, and children with the ADRA2A rs553668 GG/GA genotype were more likely to develop ADHD than those with the ADRA2A rs553668 AA genotype.

### 2.2 ADHD environmental risk factors

Environmental risk factors, which mostly include psychosocial variables, pregnancy, and perinatal risk factors, are directly linked to the development of ADHD.

#### 2.2.1 Pregnancy and perinatal risk factors

The development of children with ADHD has been discovered to be influenced by perinatal circumstances, environmental variables, intrauterine factors, and maternal self-factors throughout pregnancy. For example, children are more likely to acquire ADHD if their mothers smoke, drink excessively, or are exposed to air pollutants (polycyclic aromatic hydrocarbons), or field nonionizing nonionizing radiation when they are pregnant (33–36). Acetaminophen exposure during pregnancy has been reported to be highly related to a high incidence of ADHD in children (37). There have also been reports of possible risk factors for ADHD in children, including maternal pre-pregnancy overweight or obesity, severe mental illness in the parents, hypothyroxinemia, depression, and gestational diabetes mellitus (38–43).

#### 2.2.2 Psychosocial factors

Psychosocial factors primarily include parental mental health, marital harmony, parental relationships, and family education methods. However, the impact of the natural physical environment, including the children's living conditions and family's financial status on the onset of symptoms and overall course of ADHD patients should not be discounted. For instance, Nilsen et al. (44) discovered that children who are exposed to cigarette smoke may be more likely to experience symptoms of ADHD. The financial status of a family may also effect a child's likelihood of developing ADHD (45). Low family income has often been linked to an elevated risk of ADHD in children within research (46, 47). Meanwhile, the development of ADHD can also be influenced by parental mental health, marital status, and parental relationship status. A study (48) confirmed that mothers of ADHD children exhibit more pronounced symptoms of anxiety and depression than their fathers. Furthermore, children with ADHD are frequently exposed to inappropriate educational techniques for extended periods, which makes them increasingly likely to exhibit abnormal behavioral patterns.

Scholars have studied the mechanisms by which environmental factors contribute to the development of ADHD in children. It was found that children whose mothers smoked during pregnancy had reduced volumes of cortical gray matter, cerebellum, and corpus callosum, and thinning of frontal, temporal, and parietal regions, along with alterations in the white matter microstructure of several major connective bundles. Importantly these alterations in brain regions have also been associated with deficits in cognitive performance, auditory processing, social development and ADHD. At the same time, studies have shown that adolescents whose mothers smoked during pregnancy showed inefficient recruitment of relevant brain regions (including the temporal lobe, hippocampus and cerebellum) during response inhibition, attention and memory tasks (49). Prenatal alcohol-exposed children have reduced brain volumes and abnormalities in the frontal, parietal, and temporal lobes in terms of volume, gray matter density, shape, and cortical thickness (50), which have been associated with attention deficits and impulsive behavior (51).

Peterson et al. applied magnetic resonance imaging studies have found that prenatal exposure to PAH air pollutants disrupts the development of white matter in the left hemisphere of children, particularly in the frontal, parietal, and temporal lobes, which can lead to ADHD symptoms and externalizing problems since this white matter contributes to attention and impulse control (33). Non-clinical studies have shown evidence of various potential mechanisms for the deleterious effects of acetaminophen on neurodevelopment. Blecharz-Klin et al. (52) found that rats receiving therapeutic doses of acetaminophen significantly modulated neurotransmission in brain structures (prefrontal cortex, hypothalamus, and striatum) associated with behavior and working memory.

The contribution of maternal overweight/obesity to unfavorable brain development is partly influenced by inflammatory phenomena. Thus, inflammation may play a role in the association between maternal overweight/obesity and ADHD in children and adolescents. Obese pregnant women have higher circulating levels of pro-inflammatory cytokines than normal-weight pregnant women, and the normal developmental trajectory of the fetal brain may be interrupted by exposure to infections and high levels of pro-inflammatory cytokines, with long-lasting or persistent consequences for gray matter volume and white matter integrity (53, 54). This leaves the fetus vulnerable to psychiatric complications, and thus ADHD is associated with elevated levels of inflammatory cytokines (55, 56).

Recent data from the existing literature suggests that gestational diabetes mellitus (GDM) promotes altered structural brain morphology in the offspring. Van Dam et al. found reduced neuronal excitability and neuronal plasticity in children whose mothers had GDM, suggesting that GDM may lead to central nervous system dysfunction, which may be further associated with impaired cognitive and motor outcomes (57). In a recent study, researchers used diffusion tension imaging studies to show that infants of mothers with GDM exhibited microstructural white matter abnormalities associated with impaired neurocognitive abilities (58). Lynch et al. found that reduced hippocampal volume in specific subregions of children exposed to GDM *in utero* is accompanied by specific alterations in hippocampal morphology (59). In another recent study, Ahmed et al. observed reduced cortical thickness in multiple brain structures and poorer overall cognitive performance in diabetes-exposed offspring (60).

### 2.3 Epigenetic involvement in the pathogenesis of ADHD

In the field of genetics, epigenetics focuses on gene expression and functional regulation rather than alterations to the DNA sequence. The most popular topics of study in epigenetics include DNA methylation, histone modification and non-coding RNA (61). Neurodevelopmental disorders are diseases in which epigenetics plays a prominent role.

#### 2.3.1 DNA methylation

DNA methylation is one of the most researched types of modification in the field of epigenetics. By adding methyl groups to the DNA bases, it controls the expression of genes. Research has revealed that the DRD4 gene exhibits evidence of methylation, with varying levels of methylation among individuals diagnosed with ADHD (61). Moreover, variations in the DNA methylation of genes associated with the dopamine signaling pathway may result in modified expression levels of the relevant genes, which could impact dopamine signaling and neurodevelopment, ultimately linking them to the pathogenesis of ADHD (62, 63). In the methylome analysis of salivary DNA from children with ADHD, Wilmot et al. (64) discovered altered DNA methylation of the vasoactive intestinal peptide receptor 2 (VIPR2). Additionally, methylation of cytosine-phosphate-guanine (CpG), a crucial location upstream of DRD4, plays a significant part in dopamine's ability to regulate. Meanwhile, Wilmot also demonstrated that male children with ADHD had higher levels of CpG methylation in their peripheral tissues, indicating that DNA methylation markers in these children's peripheral tissues may be useful for research in the future. While the study by Walton et al. (65) did not find evidence of differential methylation of DRD4 and VIPR2, it discovered that potential symptomatic changes in children with ADHD have been distinguished by DNA methylation at birth at multiple genomic locations.

Additionally, early-life methylation patterns in the peroxisome network may impair the production of docosahexaenoic acid (DHA), which may contribute to the symptoms of ADHD in children and adolescents. Chen et al. (66) studied twin children with ADHD and discovered several different methylated genes between ADHD and non-ADHD siblings. In contrast to the Wilmot result of hypomethylation of the VIPR2 gene, differential methylation of the VIPR2 gene was also discovered, with hypermethylation in three afflicted twins.

#### 2.3.2 Histone modification

Another significant epigenetic mechanism that alters the modification marks on histone proteins to control gene expression is histone modification. Common histone modifications linked to gene activation and expression include acetylation, methylation and phosphorylation. They play an important role in different nuclear processes, such as replication, DNA repair, transcription, and chromatin structure stabilization (67). However, relatively little work has been done on the role of histone modifications in the pathogenesis of ADHD. Xu et al. (62) studied blood samples from Chinese Han children and found increased expression of histone deacetylase 1 (HDAC1) in children with ADHD compared to healthy controls, suggesting that protein acetylation is reduced in the group of children with ADHD. Histone methylation and acetylation abnormalities, can all result in aberrant gene expression, which can then influence neurodevelopment, synaptic function, and dopamine signaling, impacting the pathogenesis of ADHD.

#### 2.3.3 Non-coding RNAs

Non-coding RNAs are also implicated in the pathophysiology of ADHD, in addition to DNA methylation and histone modification. The expression and function of genes can be controlled by these non-coding RNA molecules (68). Notably, individuals with ADHD may have different levels of several non-coding RNAs' expression. These non-coding RNAs have the potential to impact neurodevelopment, synaptic function, and dopamine signaling pathways through controlling the expression of particular genes. Srivastav et al. (69) observed that peripheral microRNA concentrations were different in both animal models and children with ADHD. In 2023, Dypås et al. discovered 32 microRNAs that were strongly linked to features related to ADHD, including hyperactivity (29) and inattention (3) (70).

## 3 Comorbidities of ADHD

Comorbidity refers to the presence of more than one disease diagnosis in a patient, and ADHD is frequently comorbid with other disorders, both psychiatric disorders and non-mental diseases, with up to 70%–80% of individuals with ADHD experiencing concomitant psychiatric disorders throughout their lives (71), including oppositional defiant disorder (ODD), conduct disorder (CD), major depressive disorder (MDD), bipolar disorder (BD), anxiety disorders (AD), and substance use disorders (SUD). In addition, ADHD is highly comorbid with other neurodevelopmental disorders, such as autism spectrum disorders (ASD), learning disabilities (LD), and tic disorders (TD). According to recent research, non-mental disorders are very common in individuals with ADHD and greatly lower quality of life. Research on non-mental diseases that co-occur with ADHD has demonstrated a strong correlation with obesity, diabetes mellitus, sleep disorders, epilepsy, and allergic diseases (72–74). Comorbidities frequently cause children with ADHD to have significantly impaired social functioning and complicate their clinical presentation, diagnosis, and course of treatment. For this reason, it is critical to understand why comorbidity between ADHD and other diseases is so common.

Studies have revealed a biological explanation for the co-occurrence of ODD in children with ADHD. This includes a shared genetic basis involving the genes encoding androgen receptors and adrenal hormones. Additionally, it has been proposed that there is a strong correlation between serum 5-hydroxytryptamine concentrations and aggressive behavior in individuals. Furthermore, Noordermeer et al. demonstrated that children with comorbid ODD and ADHD displayed genetic variations in working memory, facial expression detection, and temporal processing, indicating neurocognitive impairment (75). According to an etiological study of children with co-occurring CD and ADHD, there may be hereditary and environmental factors that contribute to both conditions (76). The persistent weight of linked emotional and cognitive features in people with ADHD, as well as the association between psychosocial and functional stressors in daily life, may be the cause of ADHD and mood disorders and the prevalence of comorbid anxiety and SUD (77–79). Moreover, rather than being the result of exposure to these exposures, mental comorbidity may also be a direct expression of common genetic variables between ADHD, comorbid conditions, and related emotional, cognitive, and behavioral features. Fraporti et al. (80) reported that the interaction between the ADORA2A gene and the DRD2 gene affects anxiety disorders in children with ADHD. Demontis et al. discovered an association between anxiety disorders and the DRD2 gene in children with ADHD. A GWAS meta-analysis of ADHD revealed that obesity, insomnia, ASD, schizophrenia, MDD, and cannabis use disorders have significant genetic correlations with ADHD, and there is genetic overlap between them (74). Therefore, a major contributing factor to the explanation of the correlation between co-occurring features and comorbid psychiatric disorders and ADHD is genetics.

One of the most widely studied co-occurring nonmental diseases in ADHD patients is epilepsy. The prevalence of epilepsy ranges from 0.5% to 0.9% in children worldwide (81), and it is often comorbid with other psychiatric disorders, such as ASD, ADHD, and AD, with ADHD being the most common. Some researchers have also suggested that the relationship between epilepsy and ADHD is bidirectional (82) and that ADHD may contribute to an elevated risk of seizures, while chronic recurrent abnormal discharges in children with epilepsy may exacerbate symptoms of ADHD, such as poor attentional control and impulsive and hyperactive behavior. The pathogenesis of epilepsy co-occurring with ADHD is often caused by a combination of factors and may include related mechanisms such as structural brain abnormalities, genetic factors, and neurobiochemical mechanisms.

In terms of brain structure, Karalok et al. (83) reported that both children with benign childhood epilepsy with centrotemporal spikes (BECTs) and children with BECTs with comorbid ADHD showed cortical structure abnormalities (cortical area thinning), but comorbid ADHD in children with epilepsy was more strongly associated with cortical area thinning. In terms of genetic factors, the genotypic association between ADHD and epilepsy is extremely high, and this correlation can be explained by the influence of family aggregation, mainly on the maternal side, as well as by individual-specific environmental factors (84). A number of studies have suggested that epilepsy comorbid with ADHD may be due to the presence of common genetic abnormalities between the two, mainly in the IQSec2 gene, the SLC6A1 gene, the SLC9A9 gene, the Dlg4 gene, and the Vamp2 gene (85–87). In terms of neurobiochemistry, abnormalities in dopamine receptor function or dopamine overproduction in the central nervous system are currently considered to be the main mechanisms for the co-occurrence of epilepsy and ADHD.

## 4 Neuroimaging features

Based on some studies, cortical maturation in ADHD patients involves multiple regions and cortical dimensions. Primarily, this is manifested in ADHD patients as delayed developmental trajectories. Previously, Shaw et al. (88) also demonstrated that cortical maturation in ADHD patients is significantly delayed. Likewise, Qian et al. (89) found developmentally relevant delays in inhibitory and transfer functions in children with ADHD. Children with ADHD are more affected by subcortical abnormalities; the more delayed subcortical structure development, the more prominent the hyperactivity and impulsivity symptoms in the child, and the slower the cortical thinning of the prefrontal and cingulate regions (90, 91). Besides, a report published in 2021 identified that children with ADHD had smaller cerebral cortex total surface area, cortical thickness and subcortical areas (7). Simultaneously, the developmental trajectory of gray matter volume and cortical thickness is correlated with changes in symptoms of ADHD. The more severe the symptoms, the slower the brain matures (65, 92). Therefore, the characterization of the corresponding brain regions of ADHD patients in magnetic resonance imaging will be explored in terms of the following imaging techniques.

### 4.1 Functional magnetic resonance imaging

Functional magnetic resonance imaging (fMRI) primarily uses magnetic fields to measure the blood oxygen level-dependent (BOLD) response in the cerebral cortex and subcortical regions. By gathering data from participants while they are at rest and in various task-design scenarios, fMRI can indirectly reflect how the brain functions.

The resting-state fMRI describes the low-frequency fluctuations that arise spontaneously during an MRI scan while the patient's body is at rest and not thinking. The resting-state network describes the multiple brain regions where the fMRI signals are correlated with each other in the resting state. The default mode network (DMN), a significant resting-state network, is made up of brain areas that, in healthy individuals, exhibit increased activity during waking rest and deactivation with increasing attentional demands (93, 94). However, this negative correlation between the default mode network and attention will be diminished or nonexistent in ADHD patients, which could account for the reduced sustained attention brought on by default mode network-mediated attention deficiencies (95–98).

Moreover, Sun et al. (99) demonstrated that children with ADHD had more fragmented resting-state network connection patterns and delayed functional network development. Research employing resting-state fMRI has demonstrated dysfunctional connectivity in the brain regions of the dorsal anterior cingulate cortex and posterior cingulate cortex and aberrant developmental patterns in the interaction between the dorsal anterior cingulate cortex and DMN in patients with ADHD (99). Furthermore, TIAN et al. (100) discovered that children with ADHD had significantly improved functional connectivity in the dorsal anterior cingulate cortex as well as bilateral thalamus, bilateral cerebellum, and bilateral insula. Additionally, children diagnosed with ADHD exhibited reduced functional connectivity in the areas of the thalamus and basal ganglia (96).

fMRI combines many task paradigms to represent the various cognitive processes occurring in the brain. Studies on ADHD patients have revealed impairments in cognitive abilities such as working memory, sustained attention, and inhibitory function (101). Research has demonstrated (102) that when engaged in working memory tasks, individuals with ADHD exhibit decreased activity in bilateral frontal lobes, frontal-to-parietal areas and the insulae. Likewise, in comparison to normal controls, working memory-related brain regions consistently and repetitively exhibit underactivation (103, 104). Children with ADHD have also been observed to show under-activation of brain regions associated with inhibitory control in inhibitory function tasks (103, 104).

Furthermore, Christakou et al. (105) used magnetic resonance imaging to demonstrate that patients with ADHD exhibited significantly higher activation in precuneus regions but significantly lower activation in the left dorsolateral prefrontal cortex, superior parietal gyrus, and striatal-thalamic regions in an alertness task involving sustained attention. Additionally, children with ADHD mostly exhibit decreased activation of the right dorsolateral frontal-basal ganglia-thalamic-parietal network when doing tasks that target attentional processes (103, 106). Reduced frontal-striatal loop activation has also been seen in children with ADHD during sustained attention activities (107, 108).

### 4.2 Functional near-infrared spectroscopy

Functional near-infrared spectroscopy (fNIRS) utilizes near-infrared light to track variations in oxygen and deoxyhemoglobin concentrations over time. Notably, it has been used extensively in studies because it is less sensitive to motion artifacts than fMRI, safe, inexpensive, and requires less body immobilization. fNIRS is a useful tool for measuring brain activity during resting and task states. Blood flow and volume rise when brain areas are activated in response to stimuli, and this can be measured by determining the concentration of local hemoglobin (HbO), deoxyhemoglobin (HbR), or total hemoglobin (HbT). Thus, fNIRS is a valuable assessment instrument for neurodevelopmental research, particularly in analyzing the effects of interventions on children with ADHD (109, 110).

Researchers have demonstrated that resting-state fNIRS is repeatable in terms of functional connectivity and network topological characteristics (111–114). According to research conducted in 2020 by Wang et al. (115), children with ADHD had significantly lower functional connectivity and global efficiency of brain networks during the resting state when using fNIRS. Concurrently, each network node in the brain experienced corresponding changes in efficiency. The results may indicate deficiencies in reaction inhibition and information overload in vision and attention in children with ADHD, according to the dual pathway hypothesis. It was further established that functional connection networks and symptoms of ADHD are related. Besides, a negative correlation was identified between the reduced efficiency of the right somatomotor network nodes and the symptoms of hyperactivity and impulsivity. In 2021, based on a multiscale entropy study, Hu et al. found (116) that children with ADHD had lower brain signal variability in several functional brain networks (such as the default mode, frontoparietal network, attentional network, and visual network) than did healthy children, aligning with Wang's et al. (115) findings.

When examining the potential connection between alterations in brain activation and executive function in ADHD patients, fNIRS is a highly effective tool (117, 118). Meanwhile, a task-based investigation of dynamic functional connectivity in children with ADHD was carried out by Sutoko et al. (119). The frontal-cingulate-striatal-thalamic and frontal-parietal-cerebellar networks, which control working memory, attention, and inhibitory function, exhibit complicated multisystem deficits in ADHD patients.

In inhibitory control tasks, adolescents with ADHD have inferior connectivity within the inhibitory network (120) Sutoko et al. (121) discovered that children with ADHD tend to show a decreased likelihood of an advantageous connectivity state and an increased likelihood of other connectivity states in an inhibitory control task. In a 2023 study, Hou et al. (122) demonstrated that activation areas inhibiting cognitive interference ability were concentrated in the bilateral prefrontal cortex, whereas activation areas inhibiting control skills were broadly distributed in the bilateral prefrontal cortex, parietal and frontal regions, the left temporal and superior temporal cortex, the right inferior frontal gyrus, and the middle frontal gyrus. According to Inoue et al. (123), children with ADHD showed decreased prefrontal activation during a go/no-go task. Children with ADHD showed lower levels of brain activity in the left prefrontal cortex during go/no-go task, according to a 2017 study (124). Furthermore, Wu et al. (125) discovered that during a go/no-go task, children with ADHD had decreased levels of oxyhemoglobin concentration in the prefrontal brain. Prior research utilizing fMRI has also revealed that when children with ADHD do an inhibitory task, there is a significant bilateral decrease in cerebral blood flow in the prefrontal cortex region (126, 127).

In working memory tasks, the activation areas of verbal working memory in healthy individuals were primarily located in the right prefrontal cortex (PFC), particularly in the right ventral lateral prefrontal cortex (VLPFC) and dorsal lateral prefrontal cortex (DLPFC), and the activation areas of visuospatial working memory were found in the right prefrontal cortex, the frontal pole, and the left superior frontal cortex. However, the activation areas of patients with ADHD were less significant than those of healthy individuals, and the corresponding brain regions of working memory were either non-activated or weakly activated (122). The well-known working memory paradigm known as the n-back task has been used in functional neuroimaging studies of ADHD (128). Using fNIRS during the n-back task, Gu et al. (129) discovered that prefrontal complexity was lower in ADHD patients than in healthy controls. Two recent fNIRS investigations have demonstrated that when working on working memory tasks, the left DLPFC is more active in patients with ADHD (130, 131).

## 5 Future research directions

Future research on ADHD will focus on the following areas. Firstly, to more precisely clinically phenotype ADHD, it might be possible to synthesize genetic GWAS big data analysis with findings from ADHD cognitive science, brain connectivity mapping, and neuroimaging; in the future, and it might be possible to phenotype the ADHD phenotype in terms of functional impairment dimensions. Secondly, objective markers from genetics, epigenetics, and environmental factors that are strongly linked to the development of ADHD will be identified and utilized to facilitate early screening and support early diagnosis. Thirdly, neuroimaging studies of ADHD will gradually transition from MRI or near-infrared spectroscopic studies of ADHD to comprehensive studies integrating ADHD risk genes, specific cell types, developmental age-related brain networks, and neural network connectivity related to specific brain functions (cognition, emotion regulation, etc.). Additionally, they will map ADHD brain functions related to the age of onset of ADHD and its phenotypes, providing a powerful adjunct to the diagnosis of ADHD, as well as to typing, intervention and evaluation of therapeutic efficacy.

Furthermore, given the research on the mechanism of ADHD psychology and cognitive deficits, a digital, multi-scenario cognitive training system suitable for ADHD children of different ages will be developed, an accurate ADHD assessment system based on cognitive, behavioral, and emotional analyses as well as a digital cognitive and psychological intervention system will be established, and an intervention on ADHD cognitive deficits will be carried out with ADHD data-based prescriptions.

Besides, we will integrate ADHD neurological examination, cognitive and behavioral assessment, and brain network connection image analysis (resting state and task state) to assess ADHD children in all aspects, formulate individualized intervention plans, carry out related neuromodulation including repetitive transcranial magnetic stimulation, electroencephalographic biofeedback, magnetic resonance biofeedback, and transcranial direct current (DC)/alternating current (AC) stimulation, and observe the changes in the neuromodulation technology on the neural network connection and neural function, and establish reasonable and feasible neuromodulation therapeutic guidelines.

Finally, the pharmacological personalized treatment approach is a new direction for future ADHD treatment, aiming to identify biomarkers that can predict treatment response and guide personalized intervention. Based on genomics, neuroimaging and other neurological techniques to reveal the underlying biological mechanisms of ADHD, new interventional drugs can be developed to combat ADHD, which will help to target the treatment of different phenotypes of ADHD.

## 6 Conclusions

In summary, ADHD is a critically important neurodevelopmental condition in children. Through genetic GWAS analysis, epigenetic and neuroimaging studies in children with ADHD, researchers have discovered genome-wide significant loci associated with the development of ADHD, as well as thinning of gray matter thickness throughout the entire cerebral cortex, abnormalities in brain structure and function, and delayed development of neural networks in children with ADHD. This review offers potential uses in the investigation of the brain underpinnings of development, aging, and neurological illnesses.
